# Unmitigated Surgical Castration in Calves of Different Ages: Cortisol Concentrations, Heart Rate Variability, and Infrared Thermography Findings

**DOI:** 10.3390/ani11092719

**Published:** 2021-09-17

**Authors:** Luciana Bergamasco, Lily N. Edwards-Callaway, Nora M. Bello, Sage H. Mijares, Charley A. Cull, Stacy Rugan, Ruby A. Mosher, Ronette Gehring, Johann F. Coetzee

**Affiliations:** 1 Department of Clinical Sciences, College of Veterinary Medicine, Kansas State University, Manhattan, KS 66506, USA; rugan14@gmail.com (S.R.); r.gehring@uu.nl (R.G.); 2 Department of Animal Science and Industry, College of Agriculture, Kansas State University, Manhattan, KS 66506, USA; lily.edwards-callaway@colostate.edu; 3 Department of Statistics, College of Art and Sciences, Kansas State University, Manhattan, KS 66506, USA; nbello@ksu.edu; 4 Department of Animal Sciences, College of Agricultural Sciences, Colorado State University, Fort Collins, CO 80523, USA; mijaress@rams.colostate.edu; 5 Department of Diagnostic Medicine/Pathobiology, College of Veterinary Medicine, Kansas State University, Manhattan, KS 66506, USA; charley@mvsinc.net (C.A.C.); rubymosher60@gmail.com (R.A.M.); 6 Department of Anatomy and Physiology, College of Veterinary Medicine, Kansas State University, Manhattan, KS 66506, USA; jcoetzee@vet.k-state.edu

**Keywords:** calves, castration, cortisol, heart rate variability, infrared thermography

## Abstract

**Simple Summary:**

In the United States, castration is a common husbandry procedure utilized in the cattle industry. Despite castration being painful, it is commonly performed without the use of analgesia, one reason being the lack of available approved analgesics in the United States for use in alleviating pain associated with castration in cattle. Additionally, if pain mitigation is used, it is more often provided to older animals as there is a notion that younger animals experience pain to a lesser degree than older ones. The aim of this study was to characterize physiological responses to unmitigated surgical castration in calves of varying ages in terms of cortisol concentration, heart rate variability, and changes in eye temperature. Overall, our results indicate that the measured physiological responses to castration differed between age groups and changed over time post-castration. Younger calves showed a different response pattern than older calves for many of the variables measured suggesting that the response to castration-induced pain may be age-specific. For example, the youngest calves had lower cortisol and average eye temperature as compared to the oldest calves. Additionally, many variables showed a differential response to castration-induced pain, as compared with simulated castration, thus suggesting physiological indicators that could be targeted in future development and validation of analgesics for alleviation of pain associated with castration in cattle.

**Abstract:**

The objective was to characterize physiological responses to unmitigated surgical castration in calves of varying ages. Thirty male Holstein calves of three ages [<6 w (6W); 3 m (3M); 6 m (6M); *n* = 10] underwent a simulated castration treatment (SHAM) followed 24 h later by castration (CAST). For both treatments, heart rate variability, eye temperature, and cortisol were measured over time from treatment to specified end points to capture the acute response period. Interactions between treatment and age (*p* = 0.035) and time and age (*p* < 0.001) were noted for cortisol. The 6W calves had lower cortisol compared to 6M calves at SHAM and CAST. Cortisol of 6W calves decreased from peak to pre-treatment levels faster than 6M calves. An interaction between time and age was reported in squared differences of inter-beat-intervals (RMSSD; *p* = 0.02) and high-frequency power (HFP; *p* = 0.05), whereby both responses decreased in 6W calves during the sampling period which was not seen in 3M and 6M calves. Average eye temperature (AET) differed by age (*p* = 0.0018) whereby 6W calves had lower AET than 6M calves (*p* = 0.0013) regardless of treatment and time. The findings suggest that responses to unmitigated surgical castration seem to be mediated by the autonomic nervous system in an age-related manner.

## 1. Introduction

Bovine castration is a painful procedure typically performed without analgesia in the United States [1,2], although stakeholder groups do encourage the adoption of techniques to minimize pain and distress associated with the procedure [3,4,5]. One of the limitations with providing pain mitigation for castration in the United States is the lack of available FDA-approved drugs to control pain associated with castration [1,2,6]. Although there are several approved analgesics for use to control pain in cattle available in other countries, in the United States, there is only one approved drug authorized for this use (e.g., transdermal flunixin meglumine, specifically for pain related to interdigital phlegmon, i.e., foot rot; [7]). Identifying reliable and repeatable methods of pain assessment in cattle is key to informing recommendations on analgesic use for alleviating pain from common management practices, such as castration.

Pain is defined as an aversive sensation associated with actual or potential tissue damage, resulting in physiological, neuroendocrine, and behavioral changes that indicate a “stress” response [8,9]. Although castration is generally considered more painful in older calves, studies examining physiological and neuroendocrine responses to pain as a function of age are lacking. There are several physiological measures that can be used to measure the activation of these systems in response to a painful procedure such as castration in cattle. For example, blood cortisol concentration has been shown to increase in calves following castration (a selection: [10,11,12,13,14]). In addition, heart rate variability (HRV) is used to measure autonomic nervous system (ANS) regulation of cardiac function (e.g., sympathetic and vagal tone on the heart) and has been used to assess pain response in cattle associated with procedures such as castration with or without pain mitigation [15,16,17]. Another measurement used to assess pain in cattle in response to castration is infrared thermography (IRT), a noninvasive technique for visualization of a surface thermal profile. Eye temperature has been shown to be an effective tool to measure ANS activity in cattle related to castration [16,17,18,19,20].

The aim of this study was to characterize acute physiological responses to surgical castration in calves of varying ages without pain control (i.e., unmitigated) in terms of cortisol concentration, HRV, and changes in eye temperature. This is critical information needed to support evidence-based recommendations of pain control for husbandry practices in the United States. We hypothesize that unmitigated castration would affect these indicators in an age-dependent manner.

## 2. Materials and Methods

Research reported here was part of a large study reported in a companion paper [21] and therefore the experimental design and sampling methods are as described in that study. This research was approved by the Institutional Animal Care and Use Committee at Kansas State University (Protocol #2831). This study was a component of a federally funded grant (USDA-CSREES NRI Award No. 2009-65120-05729) exploring differences in pain response in varying ages of cattle.

### 2.1. Animals and Housing

Thirty male Holstein calves of <6 weeks (6W; 52 ± 9 kg), 3 months (3M; 89 ± 5 kg), and 6 months (6M; 139 ± 11 kg) of age (10 calves per age group) were enrolled in the study during Summer 2010. All study animals were housed at the Kansas State University Beef Cattle Research Center (BCRC; Manhattan, KS, USA). Calves came from one Kansas dairy herd and were acclimated at the BCRC for 10 d prior to study initiation. All study animals received a 4-way modified-live viral respiratory disease vaccine (Bovishield Gold, Pfizer, New York, NY, USA) and oxytetracycline (Noromycin 300 LA, Norbrook Laboratories Station Works, Newry, Co.Down N. Ireland; 9 mg/kg bodyweight IM).

Pre-weaned 6W calves were housed in individual wire-panel enclosures (1.6 m × 5.3 m). All enclosures were under a roof and hutches were not provided. Calves did have contact with other calves across the wire-paneling. Calves were bottle-fed milk replacer (Maxicare, Land O’Lakes, Animal Milk Products Co. 039, Shoreview, MN, USA) and provided *ad libitum* water and starter ration (Herd Maker Supreme B90, Land O’Lakes, Animal Milk Products Co. 039, Shoreview, MN, USA). The 3M and 6M calves were housed by age group on outdoor concrete pads (9.8 m × 18.3 m). Enclosures had a partial roof and straw bedding. Calves were provided with water and grass hay ad libitum in addition to receiving a grain-based supplement provided at 3–4 kg/head/day. To assist with subsequent data collection, calves were restrained with a rope halter, head gate, and girth straps for 30 min daily during the acclimation period.

### 2.2. Jugular Catheterization

Jugular catheters were placed following the methods of Bergamasco et al. [21]. In brief, the area over the jugular vein was clipped and 70% isopropyl alcohol and povidone iodine swabs were used to disinfect the area. Prior to performing a small skin incision to facilitate placement of a 14 G x 130 mm extended use catheter (MILACATH^®^, MILA International, Florence, KY, USA), the site was infiltrated with 2% lidocaine injection (Lidocaine Hydrochloride Injection, USP (2%) (20 mg/mL)**,** Hospira Inc, Lake Forest, IL, USA). The catheter was sutured in place using #3 nylon suture (Braunamid^®^, Braun, Bethleham, PA, USA). A heparin saline flush containing 3 USP units heparin sodium/mL saline (Heparin Sodium Injection, Baxter Healthcare, Deerfield, IL, USA) was used to maintain catheter patency.

### 2.3. Experimental Procedure

Each calf was submitted to two experimental procedures: simulated castration (SHAM) and surgical castration (CAST). The CAST treatment was performed 24 h after the SHAM treatment for each calf. Within each age group, animals were blocked by bodyweight and scrotal circumference. Processing date and an order of processing within the day were randomly assigned to each animal to avoid confounding effects. Both SHAM and CAST procedures were conducted between 0600 and 1030 on each processing day at 45 min intervals. Each calf was restrained in the chute with head movement limited by a halter drawn close to a table attachment (For-most Livestock Equipment, Hawarden, IA, USA) for approximately 30 min in order for experimental procedures to be completed; additional measurements to what is reported in this manuscript were taken [21]. The same operator performed all surgical procedures. For both treatments, the scrotum was washed with chlorhexidine disinfectant. The SHAM and CAST experimental procedures followed Bergamasco et al., [21]. For SHAM, the testes were firmly grasped, and ventral traction was applied for approximately 20 s. For CAST, the lower one-third of the scrotum was cut with a sharp, disinfected scalpel and the testes and spermatic cords were exteriorized followed by manual traction until the spermatic cord and connective tissue ruptured. This time point is indicated as “treatment” in the study timeline. After the experimental procedures, calves were returned to their home pens with access to feed, water, and rest. Calves were monitored frequently during post-castration data collection for pain for 8 h following surgery, and then twice daily for 7 d. Per the animal care and use protocol, animals were checked for signs of excessive pain based on the evaluation of the attitude, gait and posture, appetite, lying and scrotal swelling. Pain mitigation was not provided for the experimental procedures.

### 2.4. Cortisol

Blood samples were collected from jugular catheters immediately prior to the treatment (time 0) and again at 5, 10, 20, 30, 40, 50, 60, 120, 240, 480 and 720 min after castration or simulated castration. Halters remained on the animals to facilitate restraint during blood collection. Blood was drawn into serum clot activator tubes (Vacuette 6 mL Z Tubes, Greiner Bio-One, Kremsmünster, Austria) and was centrifuged at 1500× *g* for 10 min within 30 min of collection. The serum was pipetted off with transfer pipettes (Graduated 3 mL Transfer Pipettes Large Bulb, Samco Scientific, San Fernando, CA, USA), stored in 2 mL cryogenic vials (Fisherbrand Cryogenic Storage Vials, Fisher Scientific, Pittsburgh, PA, USA), and frozen at −80 °C prior to analysis for cortisol. Samples were analyzed within 2 months after collection. Serum cortisol concentrations were determined using solid-phase competitive chemiluminescent enzyme immunoassay and an automated analyzer system (Immulite^®^ 1000 Cortisol, Siemens Medical Solutions, Los Angeles, CA, USA; [22,23]). The laboratory technician performing the analysis was unaware of age classification and treatment. The Cmax and the Tmax were observed directly from the data. The AUC was calculated using the trapezoidal method.

### 2.5. Heart Rate Variability

On the day of each session (SHAM and CAST), approximately 1 h prior to Time 0, calves were haltered and fitted with heart rate monitoring equipment. Heart rate was recorded continuously using Polar heart rate monitors (S810i™, Polar Electro Oy, Helsinki, Finland). The left side of the animal was shaved and ultrasound gel (Ultrasound Gel, Medline Industries Inc., Mundelein, IL, USA) was applied to facilitate electrode contact with the thorax. The transmitter and receiver were fixed to the animal using an elastic belt and cohesive flexible bandages (Fisherbrand Cohesive Flexible Bandage, Fisher Scientific, Pittsburgh, PA, USA). Heart rate monitors remained on the calves while they were in the chute (approximately 30 min) during the treatment session to capture the acute responses to treatment and were then removed. At the end of each sampling period the stored data were downloaded onto a computer for analysis. The time points used for the HRV analysis included baseline (5 min before treatment; Base), early recovery (0–5 min after treatment; R05), middle recovery (5–10 min after treatment; R510), and late recovery (10–20 min after treatment; R1020) for both SHAM and CAST sessions. Equal time periods of 5 min were analyzed to fulfill recommendations for analysis of HRV [24]. Continuous recordings of R–R (interbeat) interval data are prone to measurement errors therefore, prior to analysis, a correction function within the Polar software (Polar Precision Performance Software; Version 4.03), set on default parameters, was used to correct for any artifacts (e.g., to eliminate ectopic heartbeats). Time domain parameters included heart rate (HR) and the square root of the mean squared differences of successive inter-beat-intervals (RMSSD).

Frequency domain parameters included high-frequency power (HFP) (0.30–0.80 Hz), the low-frequency power (LFP) (0.04–0.30 Hz) and the LFP/HFP ratio, which were calculated using a parametric method based on an autoregressive model for frequency-domain analysis provided by Polar software (Polar Precision Performance Software; Version 4.03). The HFP and LFP were expressed in normalized units [as a percentage or proportion of total power (e.g., LFP/total power × 100 or HF/total power × 100) to account for inter-individual differences. The HRV data were analyzed by an operator blinded to treatment and age classification.

### 2.6. Infrared Thermography

Infrared images of the eye region were collected at a consistent distance (approximately 0.5 m) and angle (90°) from the left side of the animal using an infrared camera (ThermaCAM^®^ P65HS, FLIR Systems, Wilsonville, OR, USA). Maximum, minimum, and average temperature (°C) within the area of the medial posterior palpebral border of the lower eyelid and the lacrimal caruncle were recorded every 4 to 6 s throughout the entire treatment session while the calves were in the chute. The time points analyzed included immediately before treatment (PRE) and immediately after treatment (POST). Images were analyzed for changes in temperature using research-grade software (Thermacam Researcher Pro 2.8 SR-1, FLIR Systems, Wilsonville, OR, USA). Ambient temperature and relative humidity in the barn were recorded and entered into the infrared camera to ensure calibration for atmospheric conditions. The IRT data were analyzed by an operator blinded to treatment and age classification.

### 2.7. Statistical Analysis

A general linear mixed model was fitted to each response variable included in the study. The LFP/HFP ratios were log-transformed to ensure variance stabilization, and normalized LFP and HFP were arcsine square root transformed for similar reasons. In all models for all response variables, the linear predictor included the fixed effects of treatments (namely the experimental sessions: SHAM vs. CAST), age, time points specific to each response and all 2- and 3-way interactions. The random effect of calf nested within the age group was included in the model to recognize calf as the experimental unit for age and the blocking factor for treatment. Moreover, the random effect of calf-by-treatment combination recognized calf as the experimental unit for treatment, and the random effect of calf-by-treatment-by-time combination accounted for technical replication, where applicable. Model assumptions were evaluated using studentized residuals and were considered to be appropriately met. The models were fitted using the GLIMMIX procedure of SAS (Version 9.2, SAS Institute, Cary, NC, USA), implemented using Newton–Raphson with ridging as the optimization technique. Results are presented in the original data scale (estimated least square means and corresponding standard errors or 95% confidence intervals). Relevant pairwise comparisons were conducted using Tukey–Kramer or Bonferroni adjustments, depending on the level of inference, to avoid inflation of Type I error rate due to multiple comparisons. *p*-values at or below 0.05 were used to determine statistically significant differences. *p*-values < 0.10 were considered indicative of marginal significance. 

## 3. Results

### 3.1. Cortisol Concentrations

For cortisol concentrations, 2-way interactions were identified between treatment and age (*p* = 0.035), between time and age (*p* < 0.0001) and between treatment and time (*p* < 0.0001). Therefore, results are presented by evaluating cortisol dynamics over time after SHAM and CAST for each age group. 

During SHAM, cortisol concentration decreased from peak levels to pre-treatment levels within 50 min post-treatment in 6W calves, whereas for 3M and 6M calves, cortisol concentrations returned to baseline values 120 min after the treatment (Table 1). During CAST, cortisol concentrations for 6W and 3M calves decreased from peak levels to pre-treatment levels within 120 min after treatment, whereas for 6M calves, cortisol concentrations returned to baseline values 240 min after treatment (Table 2).

Regarding cortisol AUC, only a significant main effect of treatment was identified (*p* = 0.0071), whereby cortisol AUC was greater for calves in CAST (18,719 ± 1015 nmol/L) relative to SHAM (15,222 ± 1011 nmol/L) across all age categories. No evidence for differences in AUC was apparent between age groups (*p* = 0.1460).

For Cmax, a 2-way interaction between age and treatment was identified (*p* = 0.0184), whereby, when subjected to SHAM, 6M calves had a lower Cmax (58.23 ± 20.03 nmol/L) than 3M calves (144.17 ± 7.61 nmol/L; *p* < 0.05) but Cmax was marginally different in 6W calves (129.51 ± 18.48 nmol/L; *p* = 0.0657). Additionally, during CAST, 6M calves had lower Cmax (83.36 ± 20.03 nmol/L) than 3M calves (134.90 ± 7.61 nmol/L; *p* = 0.033) and Cmax was marginally different from 6W calves (147.56 ± 18.48 nmol/L; *p* = 0.0953). The magnitude of difference in Cmax between 6M and 3M calves in SHAM was not as great as compared to CAST.

Finally, for Tmax, a main effect of treatment was observed (*p* = 0.008), whereby Tmax was greater following CAST (26 ± 2 min) compared to SHAM (20 ± 2 min) for all age categories. No evidence for any differences in Tmax was apparent between age categories (*p* = 0.3156).

### 3.2. Heart Rate Variability

**Figure 1:**
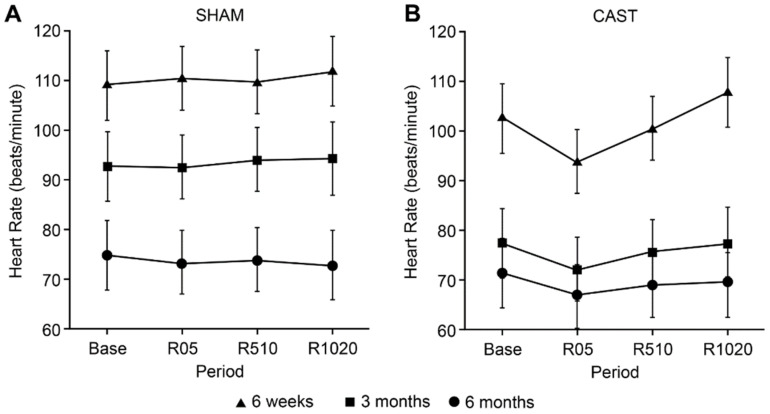
Estimated least-square means and mean standard error of heart rate (beats/min) observed during SHAM (**A**) and CAST (**B**) for 6-week (6W; triangle), 3-month (3M; square) and 6-month (6M; circle) of age. Time points evaluated consist of baseline (5 min before treatment; Base), early recovery (0–5 min after treatment; R05), middle recovery (5–10 min after treatment; R510), and late recovery (10–20 min after treatment; R1020).

When HR was evaluated, evidence for a 2-way interaction between treatment and time was found (*p* = 0.0024; Figure 1). At baseline, none of the age groups showed any evidence for differences in HR between SHAM and CAST. However, at R05 and R510, HR was lower for CAST relative to SHAM across all age groups (*p* = 0.0009 and *p* = 0.012, respectively). In addition, a significant main effect of age was identified on HR (*p* = 0.0006); whereby, regardless of SHAM or CAST, 6W calves showed greater HR (105.7 ± 5.66 beats/min) than older calves (3M: 84.33 ± 5.39 beats/min; 6M: 71.52 ± 5.39 beats/min; *p* = 0.0280 and *p* = 0.0004 respectively). No evidence for differences in HR was apparent between 3M and 6M old calves (*p* = 0.2304).

For RMSSD, there was a 2-way interaction between age and time (*p* = 0.0178), whereby for 6W calves, RMSSD decreased from the middle (R510; 68.8 ± 11.9 ms) to late (R1020; 48.8 ± 11.2 ms) recovery times (*p* = 0.0389) for both CAST and SHAM; this was not apparent in 3M and 6M calves.

**Figure 2:**
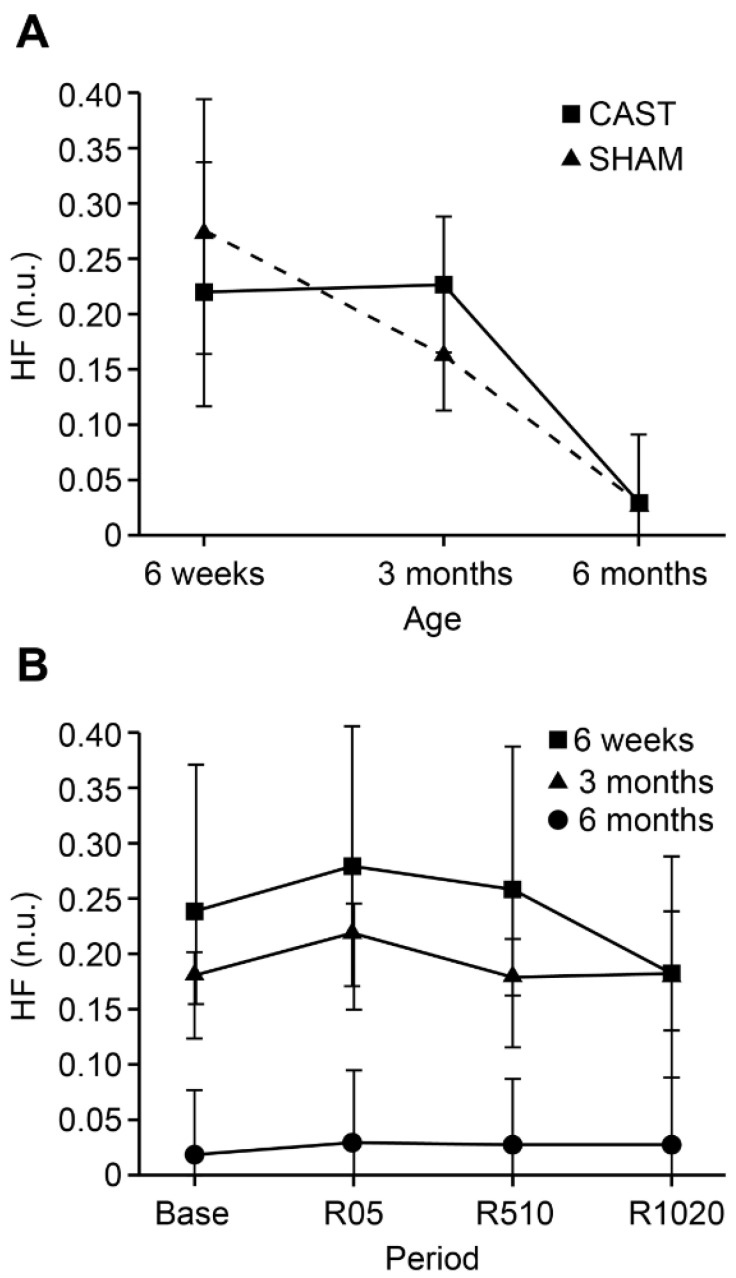
(**A**) Estimated least-square means and 95% confidence intervals of high-frequency power (HF; Hz) for 6-week (6W), 3-month (3M) and 6-month (6M) old calves undergoing CAST (square) and SHAM (triangle) (**B**) Time points evaluated consist of baseline (5 min before treatment; Base), early recovery (0–5 min after treatment; R05), middle recovery (5–10 min after treatment; R510), and late recovery (10–20 min after treatment; R1020).For LFP, there was no evidence for any differences between age groups or treatments over time. For LFP/HFP, evidence for a main effect of age was noted (*p* = 0.0059), whereby 6M old calves (estimated least square mean, [95% confidence interval]: 5.35 n.u., [2.75, 10.43]) showed greater LFP/HFP than younger calves (6W: 0.74 n.u., [0.41, 1.33], *p* = 0.0058; 3M: 1.22 n.u., [0.89, 1.67], *p* = 0.0044) regardless of SHAM or CAST.

For HFP, a 2-way interaction between treatment and age (*p* = 0.0133) was identified whereby during SHAM 6M calves had lower HFP than younger (3M and 6W) calves (*p* = 0.0126 and *p* = 0.0282, respectively; Figure 2A). During CAST, 6M calves had lower HFP than 3M calves (*p* = 0.0048) whereas the difference between 6M and 6W calves was marginally significant (*p* = 0.0613). Additionally, a 2-way interaction between age and time (*p* = 0.0517) was observed, such that 6W calves showed a decrease in HFP from R510 to R1020 (*p* = 0.0008; Figure 2B). By contrast, 3M and 6M calves showed no evidence of any change in HFP over time.

### 3.3. Infrared Thermography

For average eye temperature (AET), a 2-way interaction was apparent between treatment and time (*p* = 0.029), whereby differences in AET were identified between SHAM and CAST both before treatment (PRE; SHAM: 34.87 ± 0.26 °C; CAST: 35.41 ± 0.26 °C; *p* = 0.0179) and immediately after treatment (POST; SHAM: 34.47 ± 0.27 °C; CAST: 35.26 ± 0.26 °C; *p* = 0.001), though the POST difference was of greater magnitude. In addition, age differences were also noted on AET (*p* = 0.0018), whereby 6M calves (36.1 ± 0.4 °C) had greater AET than 6W calves (33.8 ± 0.4 °C; *p* = 0.0013) regardless of treatment and time; 3M calves had an intermediate AET (35.2 ± 0.4 °C) that was not significantly different from the other age groups.

Regarding minimum eye temperature, there was a marginally significant effect of treatment (*p* = 0.0523), such that, regardless of age, minimum eye temperature was greater during CAST (30.1 ± 0.5 °C) than during SHAM (29.1 ± 0.5 °C).

## 4. Discussion

This study characterized the acute physiological responses to unmitigated surgical castration in calves of varying ages, specifically cortisol concentration, heart rate variability, and changes in eye temperature, with the ultimate goal of supporting pain mitigation strategies for routine management practices like castration. Additional outcomes are reported in Bergamasco et al. [21], a companion paper that can be referred to for further information. Despite the fact that surgical castration is a painful procedure having both acute and chronic impacts, the use of pain control is not routinely practiced in the United States [1,2]. Surveys of producers and/or veterinarians exploring pain mitigation use for various management procedures including castration have demonstrated that analgesia use increases with cattle age [2,25]. Industry guidelines for castration procedures suggest performing the procedure at the youngest age possible to reduce stress [5]. Although responses to pain associated with castration have been shown to be age-specific [17], young calves do experience acute pain as evidenced by changes in physiological and behavioral responses to painful stimuli [8,10,21]. It is critical, as pain mitigation associated with painful husbandry procedures remains an important welfare consideration, that stakeholders understand the age-specific differences in pain response related to calf age in order to establish best practices for pain relief.

Additionally, the United States Food and Drug Administration Guidance Document 123 for the development of effectiveness data for non-steroidal anti-inflammatory drugs recommends that validated methods of pain assessment are used to evaluate a drug indicated for pain relief in the target species [26]. Our findings on changes in IRT, time and frequency domain HRV responses, and increase in cortisol concentrations found during the castration procedure in this study support the acute pain response associated with surgical castration without analgesia and could be critical for future work developing and validating effective pain mitigation drugs for castration in calves.

Cortisol is widely used to quantify acute distress associated with nociception in calves because the response magnitude (Cmax), the duration of response, and/or the integrated response (AUC) reportedly correspond with the predicted noxiousness of the animal husbandry procedure [10,11,27,28]. Current recommendations regarding the optimal age and weight at the time of castration in cattle are largely predicated on studies measuring plasma cortisol response [29]. It should be noted that cortisol concentrations may also be increased in response to the stress of handling alone [22] and therefore cortisol response to painful procedures should be considered in combination with other pain indicators. In the current study, a SHAM procedure was utilized in order to discriminate between a change in cortisol due to castration-inflicted pain from that of an overall stress response associated with handling. This design was particularly helpful in that it utilized an animal-specific benchmark to assess differences in the cortisol response to an actual painful procedure vs. simply a reaction to a potentially stressful experience with increased handling and processing.

Stafford and Mellor [30] reported peak cortisol concentrations within 30–40 min post-surgical castration. In the current study, Tmax was increased following CAST (26 min ± 1.5) compared to SHAM (20 min ± 1.5) for all age categories. The establishment of adrenocortical function occurs approximately when calves reach their 2nd week of life [31]. Knowles et al. [32] reported that plasma cortisol concentrations were very high at birth, but that concentrations decreased to levels expected in adult cattle by 27 d of age. On the contrary in the present study, 6W calves showed lower cortisol concentrations compared to older calves, both at SHAM and CAST. These results are in agreement with Ting et al. [33] and might be related to the potential developmental stage of the adrenocortical function in younger calves, as reported for preterm and low-weight infants [34,35]. These infants showed a decreased baseline cortisol concentration that has been hypothesized to be related to an inability of the hypothalamus to “recognize” stress or a failure to secrete corticotropin-releasing hormone in stressful situations [34]. During the SHAM treatment, cortisol concentrations decreased from peak to pre-procedure levels faster (50 min post-treatment) in 6W than for older calves (120 min post-treatment). During CAST, cortisol concentrations of 6W calves decreased from peak to pre-procedure concentrations within 120 min post-treatment, as reported for the 3M calves, but still faster compared to 6M calves (240 min post-treatment). However, no evidence for age differences was noted on AUC and Tmax.

Heart rate in cattle has been shown to increase during stressful events [36,37] and therefore it is used as a measure of relative stress and as an indicator of the intensity of a management procedure. It is possible to assess variation in the intervals separating consecutive heart beats using HRV measurements [15,38] and consequently obtain information on the relative involvement of the sympathetic and parasympathetic systems in the autonomic modulation of the cardiac response in livestock. Physiologic reactivity to painful stimuli is associated with intrinsic adjustments of the sympathetic and parasympathetic divisions of the ANS [39]. Studies have shown that the rise in HR as a result of pain is mostly related to an increase in sympathetic activity [40]. However, the activation of the sympathetic system may be followed by a rise in parasympathetic activity to play an antagonistic role and reestablish the homeostatic balance.

Results from the present study show that HR decreased at CAST compared to SHAM, suggesting an increase in the vagal activity. It is widely accepted that the high-frequency band (HFP; 0.15 to 0.5 Hz) of the HRV represents vagal activity, while the low-frequency component (LFP; 0.04 to 0.15 Hz) is thought to reflect both sympathetic and vagal influences. Additionally, the RMSSD is the primary time domain measure of the HRV used to estimate the high-frequency beat-to-beat variations that represent vagal regulatory activity [24]. Furthermore, at acute recovery times during the CAST treatment, HR was significantly decreased compared to SHAM; also, HR decreased in CAST between early and later recovery times. This rapid decrease in HR may be due to an adaptive response of the parasympathetic nervous system (vagal nerve) to reduce the HR. Heart rate has been used in previous studies to determine responses to castration both in studies with or without pain mitigation [14,16,41,42,43]. Decreases in HR have been reported following surgical castration of calves [41] and ring castration of sheep [36]. Schwartzkopf-Genswein et al. [41] reported significantly lower HR at 15 and 30 min after castration compared with pre-castration rates that mirror the finding from the present study. It should be noted that as with cortisol concentration, heart rate alone may not be a reliable measure of pain response as it is a non-specific measure, responding to both positive and negative events similarly. Once again, the SHAM treatment was included in order to assess the impacts of handling alone, helping to identify the changes in response variables associated with a pain-specific response during CAST.

Additionally, in the current study, mean HR was greater in younger animals compared to older animals, suggesting an increased sympathetic tone or, more likely, a physiologically higher heart rate than older calves due to increased metabolic rate [44]. Additionally, RMSSD was increased in younger calves between R05 and R1020, reflecting a shift toward more parasympathetic (vagal) dominance. The same increase was noted on HFP in 6W calves compared to older calves both in CAST and SHAM treatment, while 3M calves had an increased HFP at CAST compared to SHAM. Lastly, 6M calves have higher LFP/HFP ratio compared to younger calves. This is to be expected because normalized HFP is a contributing factor to LFP/HFP ratio. Stewart et al. [16] observed a significant increase in high-frequency power from baseline in calves castrated surgically without local anesthesia, and these data are in agreement with the results from the present study. This finding seems to support that an increase in RMSSD and HFP may imply an increase in parasympathetic activity probably associated with deep visceral pain as might occur when the spermatic cords are torn [45], as the parasympathetic nervous system acts to lower HR and carry noxious impulses from the pelvic viscera, including the testes. Von Borell et al. [15] provide a review of how heart rate variability can be used to explore autonomic nervous system function in livestock in response to stressors, such as painful procedures. Moreover, the results from the present study also indicate that calves of 6 weeks of age are capable of perceiving pain, as shown by their physiological response to painful stimuli.

Infrared thermography is a noninvasive, contactless technology, which is commonly applied to measure the heat emitted from a surface and to display the temperature distribution as an image. Specific applications for IRT in the dairy and beef industries have been described, including an automated, non-invasive system for early diagnosis of infection in cattle [18]. Gloster et al. [46] concluded that eye temperature is a useful indicator of core body temperature and that it was not affected by ambient temperature. Furthermore, the role of the autonomic nervous system in controlling the eye temperature was confirmed by a drop in eye temperature that occurred following an infusion of epinephrine [47]. Epinephrine release associated with castration causes changes in sympathetic tone, so that the adrenergic effects on cutaneous blood flow are expected to induce modifications in skin temperature that can be quantified with a thermography camera. The results from the present study show a significant decrease in average eye temperature immediately after procedure both during SHAM and CAST, although the greatest magnitude of decrease was reported at CAST. A decrease in eye temperature observed following castration of calves without local anesthetic has been attributed to sympathetically mediated alterations in blood flow in capillary beds [16]. Conversely, the anticipation of exposure to various stressors may result in a rise in core body temperature, with concomitantly reduced peripheral temperature due to peripheral vasoconstriction, termed emotional fever or stress-induced hyperthermia [48]. Interestingly, the minimal eye temperature was greater at CAST compared to SHAM. When the activation of the parasympathetic nervous system occurs, it can be expected to lower cardiac output and blood pressure, resulting in vasodilation, and an increase in eye temperature. However, the detailed mechanism for the increase that was noted in the eye temperature is still unknown.

## 5. Conclusions

It is evident, as supported by this study and others, that surgical castration causes changes in physiological outcomes in cattle indicating an acute pain response. Concerns for animal welfare are increasing awareness for the need to use pain mitigation during management procedures such as castration. However, analgesia is more often provided to older animals, likely in part due to the notion that younger animals experience pain to a lesser degree than older counterparts. This study adds to the current body of literature that demonstrates younger animals do in fact experience pain in response to castration but that depending on the physiological measure, the magnitude and direction of the response is age-specific and may differ from that of older animals. Cortisol, IRT and HRV have the potential to be used in combination with each other and with other physiological and behavioral parameters to effectively assess pain and thus substantiate the need for pain mitigation during management procedures in cattle of various ages. This information will be critical as the cattle industry continues to focus on developing effective methods of pain mitigation for painful husbandry procedures such as castration.

## Figures and Tables

**Table 1:** Estimated least square means and standard error (SEM) of cortisol concentrations (nmol/L) in SHAM treatment at the different time points (min) for the calves of 6 weeks (6W), 3 months (3M) and 6 months (6M) of age, respectively.

Time (min)	6W	3M	6M
0	23.4 ± 7.3	25.8 ± 7.2	18.2 ± 7.2
5	56.6 ± 7.3 *	116.0 ± 7.2 *	83.6 ± 7.2 *
10	60.3 ± 7.3 *	123.9 ± 7.2 *	97.3 ± 7.2 *
20	74.0 ± 7.3 *	128.3 ± 7.2 *	99.1 ± 7.2 *
30	67.8 ± 7.3 *	127.3 ± 7.2 *	98.8 ± 7.2 *
40	58.3 ± 7.3 *	105.0 ± 7.2 *	78.7 ± 7.2 *
50	48.7 ± 7.3	87.6 ± 7.2 ^‡§‖^	67.2 ± 7.2 ^‡§‖^
60	39.5 ± 7.3 §	67.4 ± 7.2 *^†‡§‖¶^	51.7 ± 7.2 *^†‡§‖^
120	11.9 ± 7.3 ^†‡§‖¶#^	22.4 ± 7.2 ^†‡§‖¶#^**	15.8 ± 7.2 ^†‡§‖¶#^**
240	5.4 ± 7.3 ^†‡§‖¶#^**	10.9 ± 7.2 ^†‡§‖¶#^**	7.8 ± 7.2 ^†‡§‖¶#^**
480	10.5 ± 7.3 ^†‡§‖¶#^	14.0 ± 7.2 ^†‡§‖¶#^**	13.2 ± 7.2 ^†‡§‖¶#^**
720	10.6 ± 7.3 ^†‡§‖¶#^	11.6 ± 7.2 ^†‡§‖¶#^**	5.3 ± 7.2 ^†‡§‖¶#^**

* Statistically different from Time 0; ^†^ statistically different from Time 5; ^‡^ statistically different from Time 10; ^§^ statistically different from Time 20; ^‖^ statistically different from Time 30; ^¶^ statistically different from Time 40; ^#^ statistically different from Time 50; ** statistically different from Time 60.

**Table 2:** Estimated least square means and standard error (SEM) of cortisol concentrations (nmol/L) in CAST treatment at the different time points (min) for the calves of 6 weeks (6W), 3 months (3M) and 6 months (6M) of age, respectively.

Time (min)	6W	3M	6M
0	14.7 ± 7.2	24.0 ± 7.2	11.2 ± 7.3
5	69.2 ± 7.2 *	95.7 ± 7.2 *	80.5 ± 7.3 *
10	79.4 ± 7.2 *	115.4 ± 7.2 *	109.8 ± 7.3 *
20	95.1 ± 7.2 *	126.4 ± 7.2 *^†^	127.5 ± 7.3 *^†^
30	93.1 ± 7.2 *	129.2 ± 7.2 *^†^	132.1 ± 7.3 *^†^
40	84.5 ± 7.2 *	111.9 ± 7.2 *	117.5 ± 7.3 *^†^
50	68.4 ± 7.2	85.4 ± 7.2 ^‡§‖^	89.3 ± 7.3 ^§‖^
60	54.4 ± 7.2 *^§^	66.6 ± 7.2 *^†‡§‖¶^	77.5 ± 7.3 *^‡§‖¶^
120	28.6 ± 7.2 ^†‡§‖¶#^	33.0 ± 7.2 ^†‡§‖¶#^**	55.0 ± 7.3 *^‡§‖¶#^
240	14.5 ± 7.2 ^†‡§‖¶#^**	22.2 ± 7.2 ^†‡§‖¶#^**	34.8 ± 7.3 ^†‡§‖¶#^**
480	8.6 ± 7.2 ^†‡§‖¶#^**	18.0 ± 7.2 ^†‡§‖¶#^**	21.0 ± 7.3 ^†‡§‖¶#^**^††^
720	16.0 ± 7.2 ^†‡§‖¶#^**	16.0 ± 7.2 ^†‡§‖¶#^**	13.6 ± 7.3 ^†‡§‖¶#^**^††^

* Statistically different from Time 0; ^†^ statistically different from Time 5; ^‡^ statistically different from Time 10; ^§^ statistically different from Time 20; ^‖^ statistically different from Time 30; ^¶^ statistically different from Time 40; ^#^ statistically different from Time 50; ** statistically different from Time 60; ^††^ statistically different from Time 120.

## Data Availability

Please contact the corresponding author with enquires about data availability.
